# Evaluation of ventricular global function from tagged CMR images

**DOI:** 10.1186/1532-429X-17-S1-Q30

**Published:** 2015-02-03

**Authors:** Abram W Makram, Ayman M Khalifa, Hossam El-Rewaidy, Ahmed S Fahmy, El-Sayed Ibrahim

**Affiliations:** 1 University of Michigan, Ann Arbor, MI, USA; 2 Helwan University, Cairo, Egypt; 3 Cairo University, Cairo, Egypt

## Background

Global measures of heart function, e.g. LV mass (LVM) and ejection fraction (EF), are usually measured from cine CMR images. On the other hand, regional measures of myocardial contractility are obtained from tagged images. The acquisition of both datasets consumes most of the CMR exam duration. In this study, we present a method for estimating measures of global heart function from the tagged images after applying a series of mathematical operations to remove the taglines and enhance the myocardium-blood contrast.

## Methods

**Figure 1:**
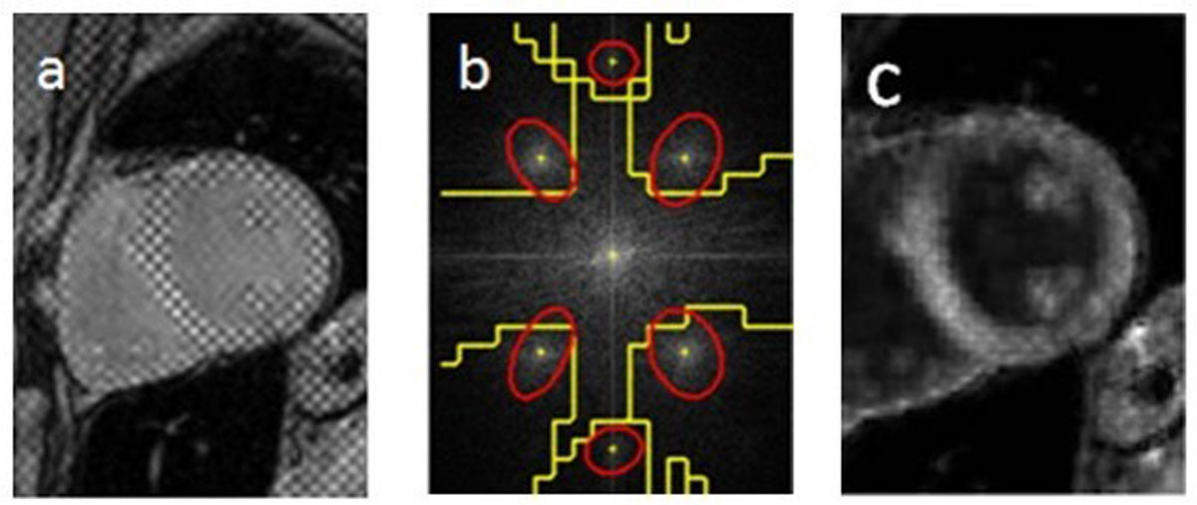
The proposed image detagging technique. (a) Originaly tagged image. (b) k-space showing automatic harmonic peaks detection (yellow boundaries dividing k-space) and the customized filters (red ellipsoids) based on PCA analysis. (c) Resulting image after applying filters and contrast enhancement.

Eleven human subjects were scanned on a 3T scanner to acquire both cine (parallel stack of short-axis slices) and tagged (basal, mid, and apical short-axis and one four-chamber slices) images covering the heart. The tagged images were Fourier-transformed (FT), and the mean-shift algorithm (MSA) method [1] was applied to each image to localize the harmonic peaks. Principal component analysis (PCA) was applied to model the harmonic energy in k-space as 2D symmetric Gaussians, which were removed using customized band-stop filters. Inverse FT was applied again to generate detagged images. Finally, local standard-deviation (LSD) [2] was calculated for each image, and multiplied by the detagged images to improve the myocardium-blood contrast (Figure 1). The endo- and epicardial contours were manually identified in the resulting images and used to estimate LVM and EF using the triangulation technique described in [3]. The results were compared to those measured from the gold-standard cine images using Diagnosoft VIRTUE software.

## Results

**Figure 2:**
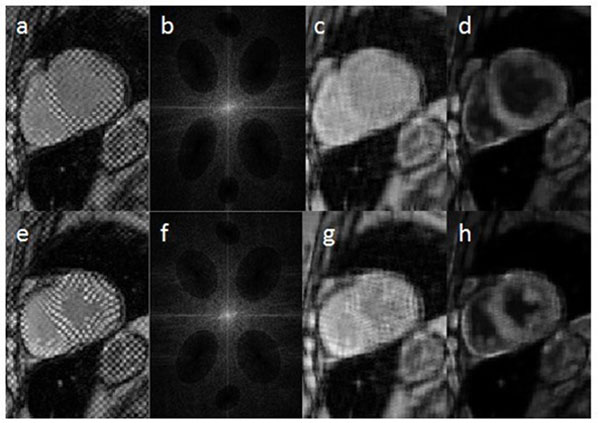
The top and bottom rows show the same mid-ventricular slice at end-diastole and end-systole, respectively. (a,e) Original tagged images; (b.f) k-spaces showing filters locations. (c,g) The resulting images after applying the band-stop filters. (d,h) Final detagged images after contrast enhancement.

Figure 2 shows short-axis mid-ventricular images at end-diastole and end-systole. The band-stop filters for the two images are slightly different as the design is optimized per each slice. The papillary muscle can be distinguished in the processed images due to the enhanced myocardium-blood contrast.

The results showed good correlation between LVM and EF measurements from the detagged and cine images. Although good agreement was observed, LVM showed stronger correlation than EF (R=0.97).

## Conclusions

A method is presented for estimating global measures of heart function from tagged cine images. The results showed good correlation with those measured from cine images. Despite the need for evaluating the proposed method on a large number of patients with different heart diseases, the developed technique would help in significantly reducing the CMR exam duration by allowing for obtaining both regional and global heart function measures from one dataset of images.

## Funding

N/A.

## References

[B1] IEEE-ISBI 2007 364 367

[B2] ISPHT 2012 1 4

[B3] IEEE-EMBC 2014 1 4 10.1109/jbhi.2015.2442632 26436157

